# A Hybrid Peptide PTS that Facilitates Transmembrane Delivery and Its Application for the Rapid *In vivo* Imaging via Near-Infrared Fluorescence Imaging

**DOI:** 10.3389/fphar.2016.00051

**Published:** 2016-03-08

**Authors:** Xuejiao Yan, Guoqiu Wu, Qingrong Qu, Xiaobo Fan, Xudong Xu, Naifeng Liu

**Affiliations:** ^1^Department of Cardiology, Affiliated Zhongda Hospital, Medical School of Southeast UniversityNanjing, China; ^2^Center of Clinical Laboratory Medicine of Zhongda Hospital, Institute of Biotechnology and Clinical Pharmacy, Medical School of Southeast UniversityNanjing, China; ^3^Department of Biological Engineering, Medical School of Southeast UniversityNanjing, China

**Keywords:** hybrid peptide, integrin αvβ3, molecular probe, tumor, near-infrared fluorescence imaging

## Abstract

**Background and purpose:** Intravital imaging provides invaluable readouts for clinical diagnoses and therapies and shows great potential in the design of individualized drug dosage regimes. Ts is a mammalian free cell membrane-penetrating peptide. This study aimed to introduce a novel approach to the design of a cancer-selective peptide on the basis of a membrane-penetrating peptide and to explore its potential as a carrier of medical substances.

**Experimental approach:**Ts was linked with a αvβ3-binding peptide P1c to create a hybrid referred to as PTS. The hybrid was labeled with an FITC or Cy5.5 as an imaging indicator to evaluate its *in vitro* and *in vivo* bioactivity.

**Key results:**Hemolysis tests proved that in comparison with Ts, PTS caused similar or even less leakage of human erythrocytes at concentrations of up to 1 mmol/L. Flow cytometry assay and confocal microscopy demonstrated the following. (1) P1c alone could target and mostly halt at the cancer cell membrane. (2) Ts alone could not bind to the membrane sufficiently. (3) P1c greatly enhanced the binding affinity of PTS with MDA-MB-231 breast cancer cells that upregulated αvβ3. (4) Ts conferred PTS with the ability to traverse a cell membrane and thus facilitate the transmembrane delivery of imaging probes. *In vivo* near-infrared fluorescence (NIRF) imaging demonstrated that the imaging probes were rapidly concentrated in a MDA-MB-231 tumor tissue within 1 h after intravenous injection.

**Conclusions and implications:**PTS exhibited the capability of targeting specific tumors and greatly facilitating the transmembrane delivery of imaging probes.

## Introduction

Molecular imaging provides anatomical and functional/molecular information that facilitates the *in vivo* diagnosis and monitoring of cancer (Liu et al., 2010; Barile et al., 2014). Existing imaging approaches consume hours or even days, and the corresponding preparations are relatively complicated (Conway et al., 2014; Yi et al., 2014). Fluorescence-based imaging approaches have been highlighted in the last decades, but their application remains constrained by the difficulty involved in inducing excitation from the inside and given an intense background. The emerging near-infrared fluorescence (NIRF) imaging technique offers a revolutionary solution to the aforementioned problems (Yi et al., 2014).

Tumor-specific cell-surface receptors allow cancer-selective targeting for tumor diagnosis and therapy (Teesalu et al., 2013; Reissmann, 2014). In a previous study, we screened a fragment peptide P1c from human connective tissue growth factor (CTGF). P1c could specifically be bound to the human integrin αvβ3. After being coupled with ultrasuperparamagnetic iron oxide particles (USPIOs), P1c was successfully applied in the magnetic resonance imaging (MRI) of a human primary liver cancer BEL-7402 that upregulated αvβ3 expression (Wu et al., 2011b).

The upregulation of integrin in tumors and neovasculature allows the preferential targeting of peptides (Mathejczyk et al., 2011; Cao et al., 2012). Cell-penetrating peptides (CPPs) are known as promising carriers of fluorophores or poorly soluble drugs for delivery across lipid membranes (Andreev et al., 2007; Reissmann, 2014). However, anticancer CCPs feature unfavorable potential for mammal cells. As an important group of CCPs, antimicrobial peptides (AMPs) derived from synthetic or natural proteins are generally cationic peptides and regarded as nontoxic to mammal cells. They preferentially target bacterial lipid bilayers because of their electrical interactions with the oppositely charged components of bacterial membranes (Reissmann, 2014). AMPs facilitate membrane intercalation and transmembrane transportation through a holing mechanism at cytoplasmic membranes (Rousselle et al., 2000; Duchardt et al., 2009). The holing/membrane penetration process is prompt and is usually accomplished within several minutes (Rahnamaeian, 2011; Fan et al., 2013). Integrating CCPs with a drug delivery feature could greatly facilitate the rapid accumulation of medical substrates in cells. Ts, which is an AMP with an antiparallel β-sheet constrained by disulfide bonds, shows selectivity on bacterial cell membranes, but its cytotoxicity is limited for mammalian cells (Fehlbaum et al., 1996; Wu et al., 2010, 2011a).

A hybrid system that constitutes a tumor-preferred peptide and a nontoxic membrane-penetrating peptide shows great potential for cancer-specific delivery. In the present study, a hybrid PTS peptide consisting of Ts following at the N-terminal of P1c was designed to combine the merits of P1c and Ts. The *in vivo* and *in vitro* activities of this hybrid system were evaluated via flow cytometry, confocal laser scanning microscopy, and NIRF imaging.

## Materials and methods

### Materials and reagents

#### Preparation of the fluorescence probe

The peptides and their conjugates were all obtained from Scipeptide (Shanghai, China) as indicated in Table 1. The peptides were synthesized with a solid-phase method using a model 432A synthesizer (Applied Biosystems Inc., Foster City, CA; Angiolillo et al., 2004). Cy5.5-NHS or FITC was dropped into the PTS, P1c, or Ts solution containing 0.1 mol/L sodium carbonate-sodium bicarbonate (pH 9.0) at an equal molar ratio. After overnight stirring in the darkness at 4°C, the reaction was blocked by adding an ammonium chloride solution to achieve a final concentration of 50 mmol/L. The crude product was purified with HPLC with a C18 Delta-Pak column (Waters, Bedford, MA) using a 0–60% acetonitrile gradient in 0.05% trifluoroacetic acid as the mobile phase. The elution containing Cy5.5-PTS, Cy5.5-P1c, Cy5.5-Ts, FITC-PTS, FITC-P1c, or FITC-Ts conjugates was lyophilized and stored at −20°C.

**Table 1:** **The amino acid sequence and chemical composition of peptides and their conjugates**.

**Peptide**	**AA sequence**	**Purified by HPLC (purity>95%)**	**MW (kD)**	**Conjugate**	**composition^a^**	**Purified by HPLC (purity>95%)**	**MW (kD)**	**Conjugate**	**composition^a^**	**Purified by HPLC (purity>95%)**
TS	SKKPVPIIYCNRRSGKCQRM	Yes	2419.2	FITC-TS	(FITC)_1_(TS)_1_	Yes	2921.2	TS-Cy5.5	(TS)_1_(Cy5.5)_1_	Yes
P1c	CIRTPKISKPIKFELSG	Yes	1916.3	FITC-P1c	(FITC)_1_(P1c)_1_	Yes	2419.8	P1c-Cy5.5	(P1c)_1_(Cy5.5)_1_	Yes
PTS	GSKKPVPIIYCNRRSGKCQRMGSIRTPKISKPIKFELSG	Yes	4359.6	FITC-PTS	(FITC)_1_(PTS)_1_	Yes	4861.7	PTS-Cy5.5	(PTS)_1_(Cy5.5)_1_	Yes

a *The composition indicated the molecule formula of each conjugate, for example, (FITC)_1_(TS)_1_meant the conjugate was consisted of one FITC conjugated with one TS molecule*.

#### Circular dichroism (CD) spectroscopy

To confirm the secondary structure of PTS, P1c, and Ts, CD measurement was performed with a Jasco-810 spectropolarimeter (JASCO, Tokyo, Japan) using a quartz cell with a path length of 1 mm at 25°C. The peptides were dissolved in 50% (v/v) trifluoroethanol (TFE) at 200 μmol/L for all the measurements.

#### Hemolysis assay

Human erythrocytes from a healthy adult were washed thrice with an isotonic PBS buffer (pH 7.4) and then re-suspended in four times the volume of the PBS before usage. Different concentrations of PTS, P1c, or Ts were then added and thoroughly mixed. Sterile PBS and 0.2% Triton X-100 were used as negative and positive controls, respectively. After incubation at 37°C for 15 min, the mixture was centrifuged at 10,000×g for 1 min. The supernatant was retrieved and measured for OD350 with a spectrophotometer. The percentage of hemolysis was calculated using the following equation: the percentage of hemolysis (%) = (*A*−*A*_*B*_)/(*A*_*P*_−*A*_*B*_) × 100. *A* is the OD350 absorbance of a tested sample added with peptide, and *A*_*B*_ is the absorbance of a negative control without peptide addition, whereas *A*_*P*_ is the absorbance of a positive control added with 0.2% Triton X-100.

#### Flow cytometry

Human Breast Cancer MDA-MB-231 cells or Human Embryonic Kidney 293 cells were pre-cultured with and without 5 μmol/L free P1c peptide or 2 mg/L anti-human αvβ3 monoclonal antibody at 37°C for 1 h. Afterward, the cells were harvested after being rinsed thrice with PBS. FITC-PTS, FITC-P1c, or FITC-Ts was then added to a final concentration of 2.5 μmol/L followed by an incubation of 30 min at 37°C in darkness before the conduct of flow cytometry (Becton Dickinson, San Jose, CA, USA).

The expression of αvβ3 in the MDA-MB-231/HEK293 cells was effectively evaluated via flow cytometry. The cells were briefly incubated with a 2.5 mg/L anti-human αvβ3 integrin monoclonal antibody in PBS with 1% FBS for 1 h at 4°C. The cells were then harvested and washed twice, followed by a 30 min incubation with 1 mg/L goat anti-mouse antibody-FITC at 4°C. Finally, the cells were carefully washed and fixed with 4% formaldehyde in PBS before undergoing flow cytometry analysis. A mouse IgG was used as an isotype control.

#### Confocal microscopy and colorimetric assay

MDA-MB-231/HEK293 cells were seeded and grown on a 35 mm cell culture dishes for 24 h. Thereafter, the cells were carefully washed with PBS and then incubated with 2 μmol/L of FITC-P1c, FITC-PTS, or FITC-Ts for 30 min in darkness. The cells were then washed twice with PBS, fixed with 4% formaldehyde in PBS, and finally examined with a confocal laser scanning microscope (OLYMPUS-FV1000, Japan). Some cells were pre-incubated with 4 μmol/L free P1c peptide or 2 mg/L anti-human αvβ3 monoclonal antibody before the addition of FITC-PTS to determine the bioactivity of P1c moiety in PTS.

#### Tumor xenografts model

Animal experiments were performed according to a protocol approved by the Animal Care and Use Committee of Southeast University, China. Female BALB/C nude mice (4–6 weeks old) were obtained from the Experimental Animal Center of Yangzhou, China and reared in the Animal Environmental Control Unit. Each nude mouse was subcutaneously inoculated with approximately 5×10^6^ MDA-MB-231 cells via the left foreleg. When the tumors reached 0.4–0.6 cm in diameter (10–14 days after implant), the mice were subjected to *in vivo* NIRF imaging studies.

#### *In vivo* NIRF imaging study

Mice were anesthetized through an intraperitoneal injection of pentobarbital sodium at a dosage of 30 mg/kg. The mice were weighted and grouped (*n* = 3) to ensure that no considerable difference in weight appears between groups. Primary experiments had determined that dosages below 0.2 nmol/mouse resulted in weak signals, whereas those above 5–25 nmol/mouse provided comparable signals. Therefore, Cy5.5-PTS, Cy5.5-P1c, or Cy5.5-Ts at a dosage of 5 nmol/mouse was administered via tail vein injection in this experiment. The images at various time points were photographed with the MaestroTM *In-Vivo* Imaging System (CRi, Woburn, MA, USA). The orange filter (excitation, 586–631 nm; emission, 645 nm longpass) was adopted. The tunable filter was automatically stepped in 10 nm increments from 640 to 820 nm while the camera captured images at each wavelength interval with a constant exposure time period. The spectral fluorescence images consisting of autofluorescence spectra and Cy5.5 spectra were obtained and then analyzed on the basis of their spectral patterns using the Maestro 2.10.0 software (CRi, Woburn, MA, USA). To determine the tumor contrast, the mean fluorescence intensities of the tumor (T) area at the left shoulder and the corresponding normal tissue (N) area at the right shoulder were calculated.

#### H&E and immunohistologic staining

After NIRF imaging, the mice were sacrificed, and the subcutaneous tumor tissues were completely removed before being fixed with glutaraldehyde at 4°C overnight, followed by paraffin embedding and slicing into sections of 4 μm. For H&E staining, the sliced sections were dewaxed with xylene and washed successively with ethanol and water. After hematoxylin staining for 5 min, the sections were washed with distilled water, differentiated with ethanol for 30 s, soaked for 5 min in 50°C water, and then counterstained in an eosin solution for 2 min. In the immunohistologic staining, the sections were kept in a microwave oven at 92–98°C for 15 min for antigen retrieval and then incubated with a 10% normal goat serum. The sections were then incubated with 4 mg/L anti-human αvβ3 monoclonal antibody and then with a horseradish peroxidase (HRP)-conjugated goat anti-mouse IgG (1:5000).

#### Data processing and statistics

All results were expressed as mean ± standard deviation (SD). One-way analysis of variance and student's *t-*test were performed to compare the differences between groups. Values of *P* < 0.05 were regarded as statistically significant.

## Results

### CD analysis of peptide structure

**Figure 1:**
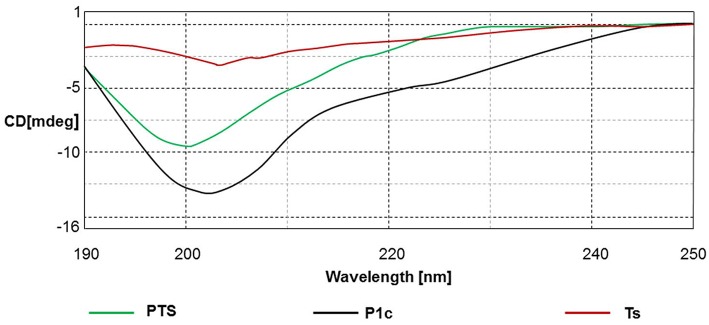
**The CD spectra of the peptides in TFE/H2O buffer (1:1, v/v)**.

The CD spectrum indicated the secondary structure of the peptides, and the results are shown in Figure 1, Table 2. PTS and Ts were prone to adopt a β-sheet structure in TFE/H2O buffer (1:1, v/v), whereas P1c did not exhibit a 2D structure. PTS featured a ~40% β-sheet corresponding to ~15 amino acids, similar to Ts that featured a ~60% β-sheet corresponding to 12 amino acids.

**Table 2:** **The secondary structure of peptides by CD spectra**.

	**Helix (%)**	**Beta (%)**	**Turn (%)**	**Random (%)**	**Total (%)**
PTS	0	40.5	5.9	53.6	100
P1c	0	0	8.3	91.7	100
Ts	0	60.8	5.1	34.1	100

### Hemolysis toxicity assay

**Figure 2:**
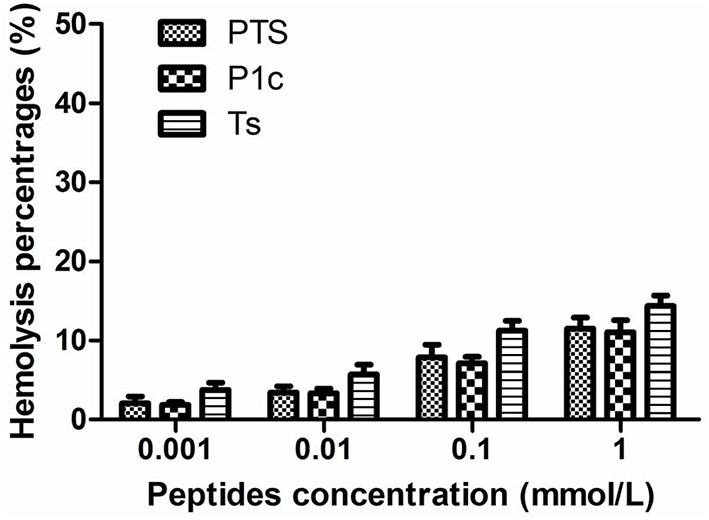
**Hemolytic toxicity of PTS, P1c, and Ts**. Human erythrocytes were incubated in phosphate buffer with various concentrations of peptides at 37°C for 15 min. There was no significant difference between three groups at tested concentrations (*P* > 0.05).

Figure 2 shows that Ts, P1c, and their hybrid exhibited similar hemolytic activities at the same molar concentrations (*P* > 0.05). In all the tested groups, hemolysis was <15% at concentrations of up to 1 mmol/L. This result indicated that the peptides were relatively safe when applied *in vivo*. In addition, Ts maintained its bioactivity without an increase in cytotoxicity after being conjugated with P1c.

### Fluorescent probes binding to MDA-MB-231 cells

**Figure 3:**
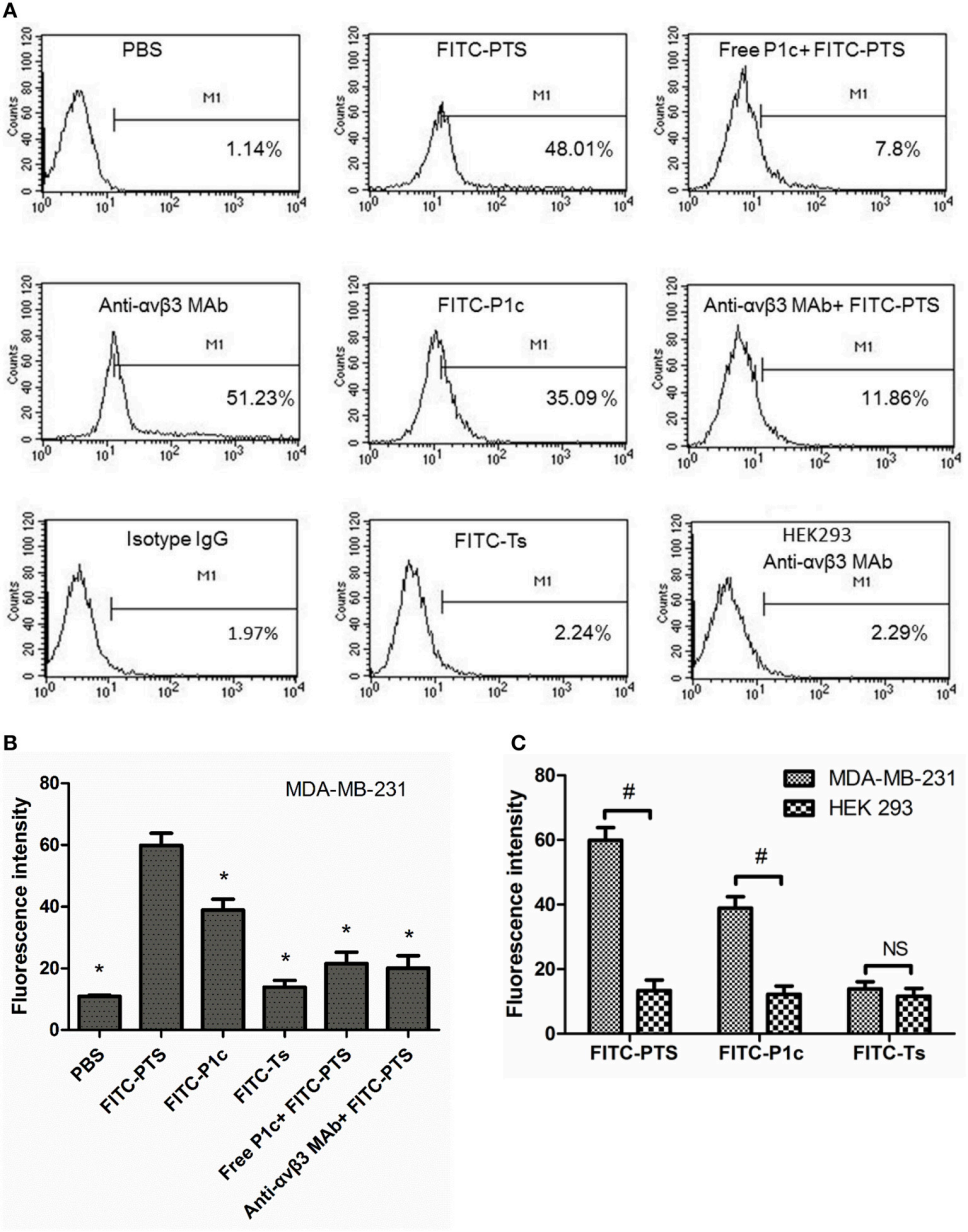
**Interaction between fluorescent probes with cells by flow cytometry. (A)** The MDA-MB-231 cells were treated with PBS, anti-αvβ3 monoclonal antibody followed by goat anti-mouse antibody-FITC, a mouse IgG followed by goat anti-mouse antibody-FITC, FITC-PTS, FITC-P1c, FITC-Ts, free P1c peptide followed by FITC-PTS, anti-αvβ3 monoclonal antibody followed by FITC-PTS, separately (*n* = 3), and the HEK293 cells were treated with anti-αvβ3 monoclonal antibody followed by goat anti-mouse antibody-FITC. **(B)** The fluorescent intensity of the MDA-MB-231 cells after receiving PBS or fluorescent probes. **(C)** The fluorescent intensity of the MDA-MB-231 cells and HEK231 cells after receiving FITC-PTS, -P1c, and -Ts. ^*^P < 0.05 compared to FITC-PTS group. ^#^*P* < 0.05 compared to each other.

As shown in Figure 3, the flow cytometry analysis revealed that MDA-MB-231 cells were positively expressing αvβ3 (Figure 3A, anti-αvβ3 MAb group) and that the signal of cells stained with FITC-PTS or FITC-P1c was significantly improved compared with that of the PBS control group or Ts group (*P* < 0.05, Figures 3A,B, Figure S1). The staining signal was significantly blocked by the addition of free P1c or anti-human αvβ3 monoclonal antibody (*P* < 0.05, Figures 3A,B, Figure S1).

**Figure 4:**
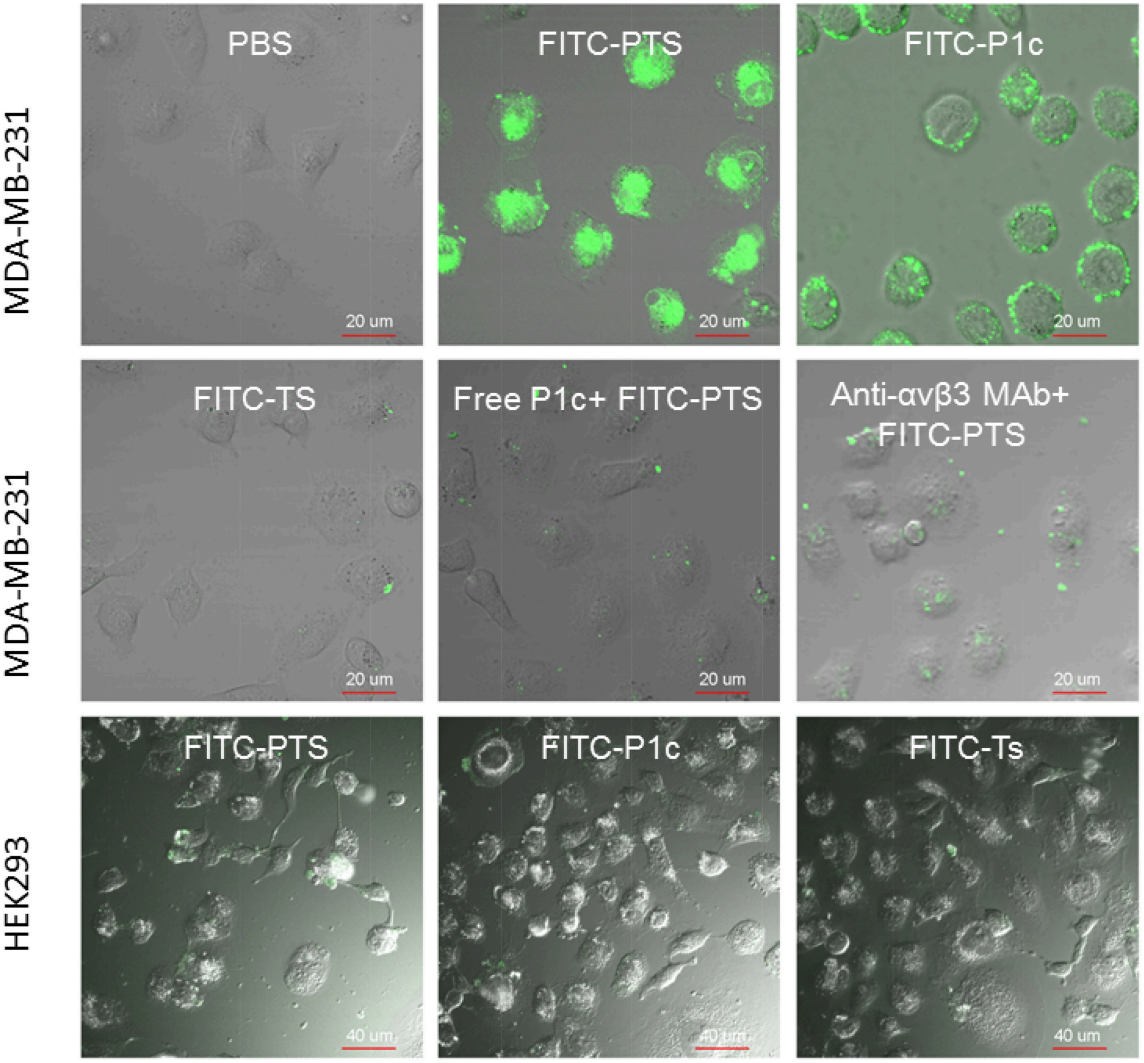
**Cellular localization of fluorescent probes**. The merged view of laser confocal florescence microscopy of MDA-MB-231 or HEK293 cells treated for 30 min with FITC-PTS, FITC-P1c, FITC-Ts, free P1c peptide followed by FITC-PTS, and anti-αvβ3 monoclonal antibody followed by FITC-PTS, separately.

As shown in Figure 4, no fluorescence appeared inside the cytoplasma, but only the cell membrane exhibited strong fluorescence after receiving FITC-P1c. By contrast, FITC-PTS was capable of transmembrane translocation and equal distribution in all the cells. This transmembrane delivery could also be prohibited by blocking αvβ3, which was upregulated at the cancer surface. Fluorescence intensity markedly decreased when the cells were pretreated with P1c peptide or anti-αvβ3 monoclonal. P1c and anti-αvβ3 monoclonal antibody could compete with FITC-P1c and FITC-PTS for αvβ3 docking sites. HEK293 cells with little αvβ3 expression (Figure 3A, HEK293 anti-αvβ3 MAb group) were much less attractive for FITC-PTS and -P1c probes (Figure 3C, Figures S2, S4).

HEK293 cells were used as a control and αvβ 3 was expressed at low level below 1% in HEK293 cells. FITC-P1c and FITC-PTS showed none affinity to HEK293 cells. In contrast to P1c and PTS, Ts and its conjugates showed none affinity to both the MDA-MB-231 and HEK293 cells.

### *In vivo* fluorescence imaging

**Figure 5:**
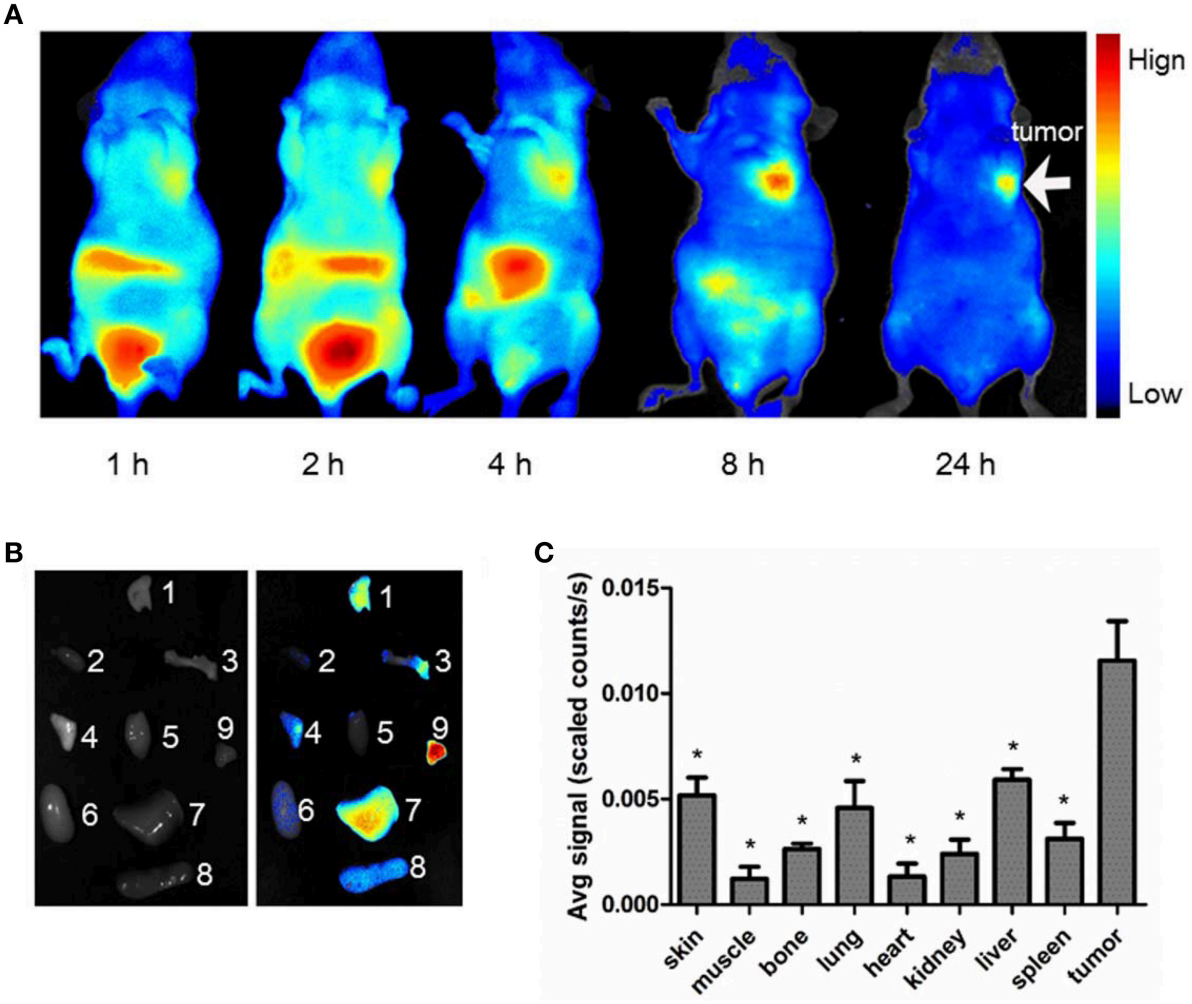
*****In vivo*** NIRF imaging**. The average signal counts were quantified for each sample/area and plotted into different colors. **(A)** Typical NIRF images of nude mice bearing subcutaneous MDA-MB-231 breast tumor after intravenous injection of Cy5.5-PTS from 0 to 24 h. **(B)** NIRF images of dissected organs from model animal at 8 h after intravenous injection of Cy5.5-PTS (left, bright field; right, NIRF imaging) and **(C)** the average scaled counts of dissected tissues. The tumors, major tissues, and organs were dissected 8 h post-injection for imaging. 1, skin; 2, muscle; 3, bone; 4, lung; 5, heart; 6, kidney; 7, liver; 8, spleen; 9, tumor. This experiment was repeated for three times with 2~3 mice for each time. ^*^P < 0.05 compared to tumor tissue.

**Figure 6:**
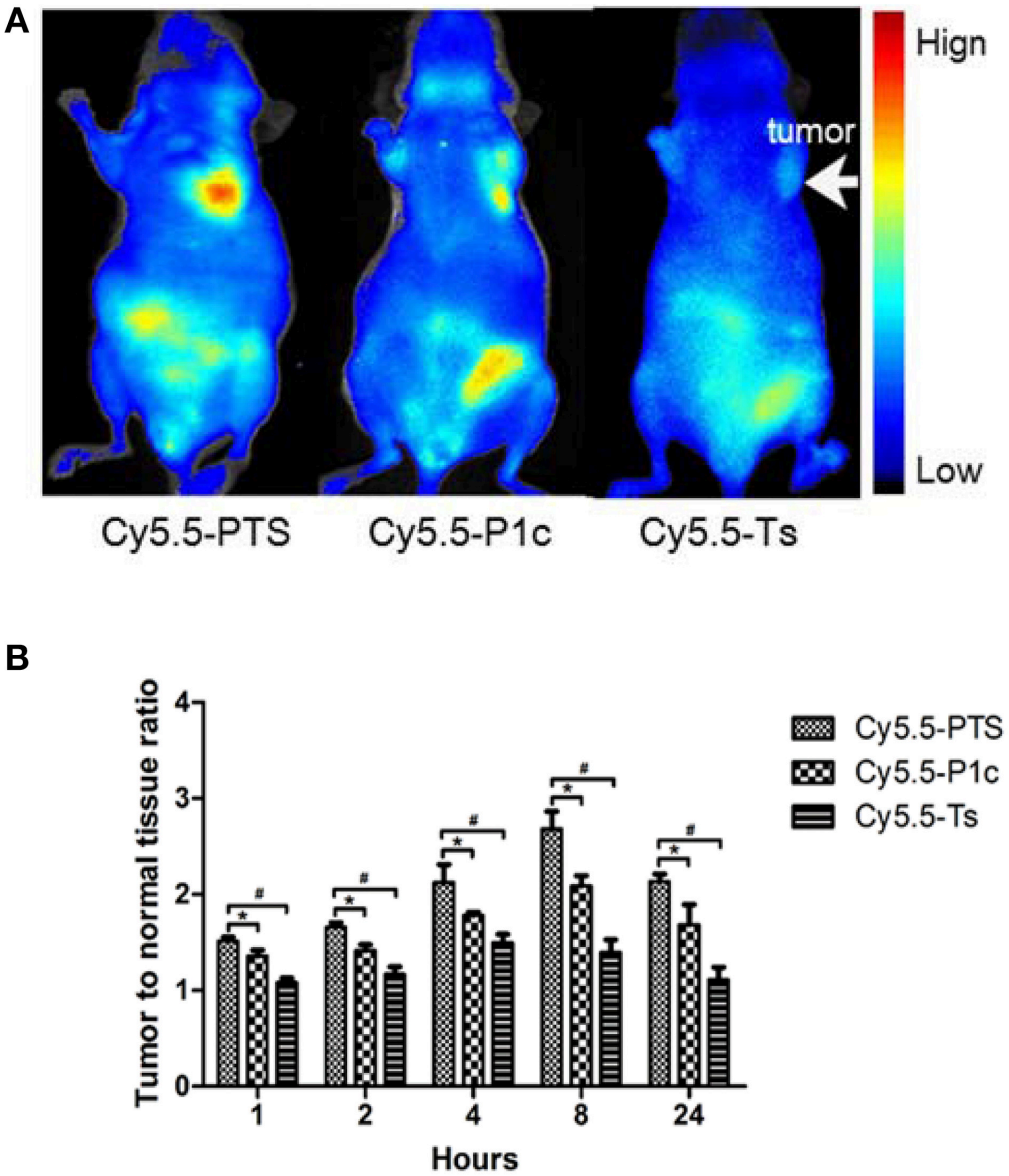
*****In vivo*** NIRF imaging**. **(A)** Representative NIRF images obtained from model animals at 8 h after intravenous injection of Cy5.5-PTS **(left)**, Cy5.5-P1c **(middle)** and Cy5.5-Ts **(right)**, respectively. **(B)** Tumor-to-normal tissue contrast ratios of three NIRF probes at time points. ^*^P < 0.05 vs. Cy5.5-P1c group. ^#^*P* < 0.05 vs. Cy5.5-Ts group.

Figure 5A shows typical NIRF images of nude mice bearing subcutaneous MDA-MB-231 breast tumor after an intravenous injection of 5 nmol Cy5.5-PTS. Each animal became fluorescent after injection, and the subcutaneous MDA-MB-231 tumor could be clearly delineated from the surrounding background tissue from 1 to 24 h. The tumor uptake reached its maximum at 8 h (Figures 5A, 6A left) and slowly washed out over time. By contrast, normal tissue, except liver tissue, showed a rapid uptake and relatively fast clearance. The *ex vivo* evaluation of the excised organs at 8 h post-injection of Cy5.5-PTS demonstrated that the compound was predominantly uptaken by the MDA-MB-231 tumor (Figures 5B,C).

The Cy5.5-P1c probe also certainly showed potential for tumor imaging (Figure 6A middle). However, the tumor-to-normal tissue contrast ratios were significantly smaller than those of Cy5.5-PTS at time points from 1 to 24 h (*P* < 0.05; Figure 6B). In addition, a slight tumor-to-normal tissue contrast was observed in the Cy5.5-Ts group (Figure 6A, right), although the tumor-to-normal tissue contrast ratios were much smaller than those of the Cy5.5-PTS group and Cy5.5-P1c group (Figure 6B).

### Histologic evaluation

**Figure 7:**
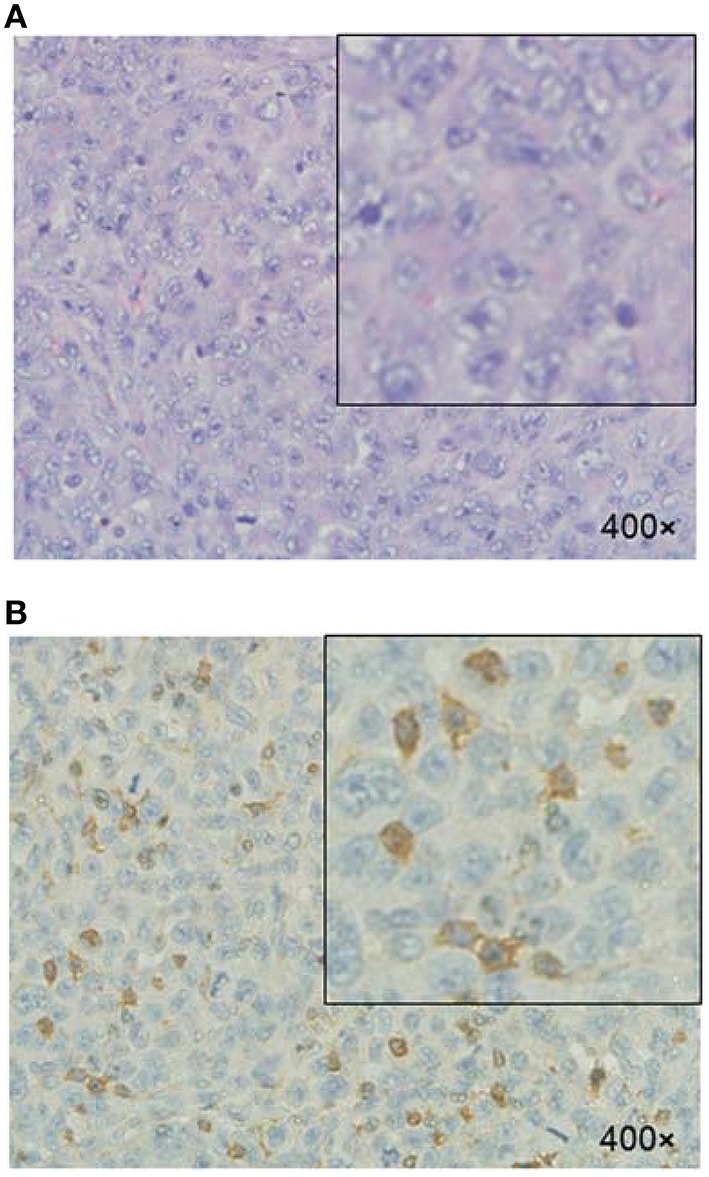
**Histologic evaluation of MDA-MB-231 tumor xenograft**. **(A)** H&E staining. **(B)** Immunohistologic staining of tumor tissues illustrated αvβ3-positive expression of tumor cells using anti-αvβ3 monoclonal antibody.

The HE staining of tumor tissue showed that the tumor cells exhibited pleomorphism and nucleus hyperchromasia. Mitotic phases and irregularly appearing nuclei were also observed (Figure 7A). The immunohistological staining of tumor tissues demonstrated that αvβ3 was expressed in the MDA-MB-231 tumor cells (Figure 7B).

## Discussion

Cell membrane-penetrating peptides employ a unique mechanism to rupture cellular membranes. Their sequence composition and charge aspects define their targeting specificity over different cellular types. Modifying their activity on the basis of sequences is extremely difficult. A single amino acid substitution or insertion would even overturn their bioactivities. In the present study, Ts was used as an example to show how to purposively design a novel peptide with distinctive activity on the basis of AMPs. The CCPs might once again become an important resource for anticancer substrates.

On the basis of our previous findings, we designed and synthesized a hybrid peptide (PTS) comprising the integrin αvβ3-binding peptide P1c linked to the C-terminal of the antimicrobial peptide (AMP) Ts. We used bacterial-preferred AMP peptides instead of anticancer CCPs because unlike anticancer CCPs, Ts would not result into an unfavorable cytotoxicity on normal cells. The anticancer CCPs showed promising anticancer activity, but their cytotoxicity was extremely high because of their surfactant resembling mechanism of action (Yeaman and Yount, 2003). AMPs were considered nontoxic to mammal cells but could not target cancer cells sufficiently. Therefore, we designed a hybrid by adding an extra αvβ3-binding peptide P1c to Ts.

This design could be extended to other combinations of tumor-targeting peptides with mammalian-cell-free CCPs. The activities of CCPs are 2D-structure dependent (Fehlbaum et al., 1996; Nguyen et al., 2011). So the combined tumor-targeting peptides should be as simple/short as possible and not affect the structure of the CCPs. A flexible linker of two amino acids was usually added between the moieties. There are numerous peptides reported of potential to target tumor-associated receptors, tissues or pathways (Raha et al., 2011), such as RGD targeting integrin (Ruoslahti and Pierschbacher, 1986), CDX-110 under developing by Celldex Therapeutics targeting EGFR (Del Vecchio and Wong, 2010), IPLVVPL targeting prostate cancer Hepsin (Kelly et al., 2008), and CREKA targeting tumor stroma (Simberg et al., 2007).

CD spectrum confirmed that PTS possessed a secondary β-sheet similar to Ts. Hemolysis assay demonstrated that PTS, Ts, and P1c exhibited similar but limited hemolysis toxicity at concentrations of up to 1 mmol/L. This result implied that Ts maintained its function in the hybrid and helped PTS to pore on the membrane.

**Figure 8:**
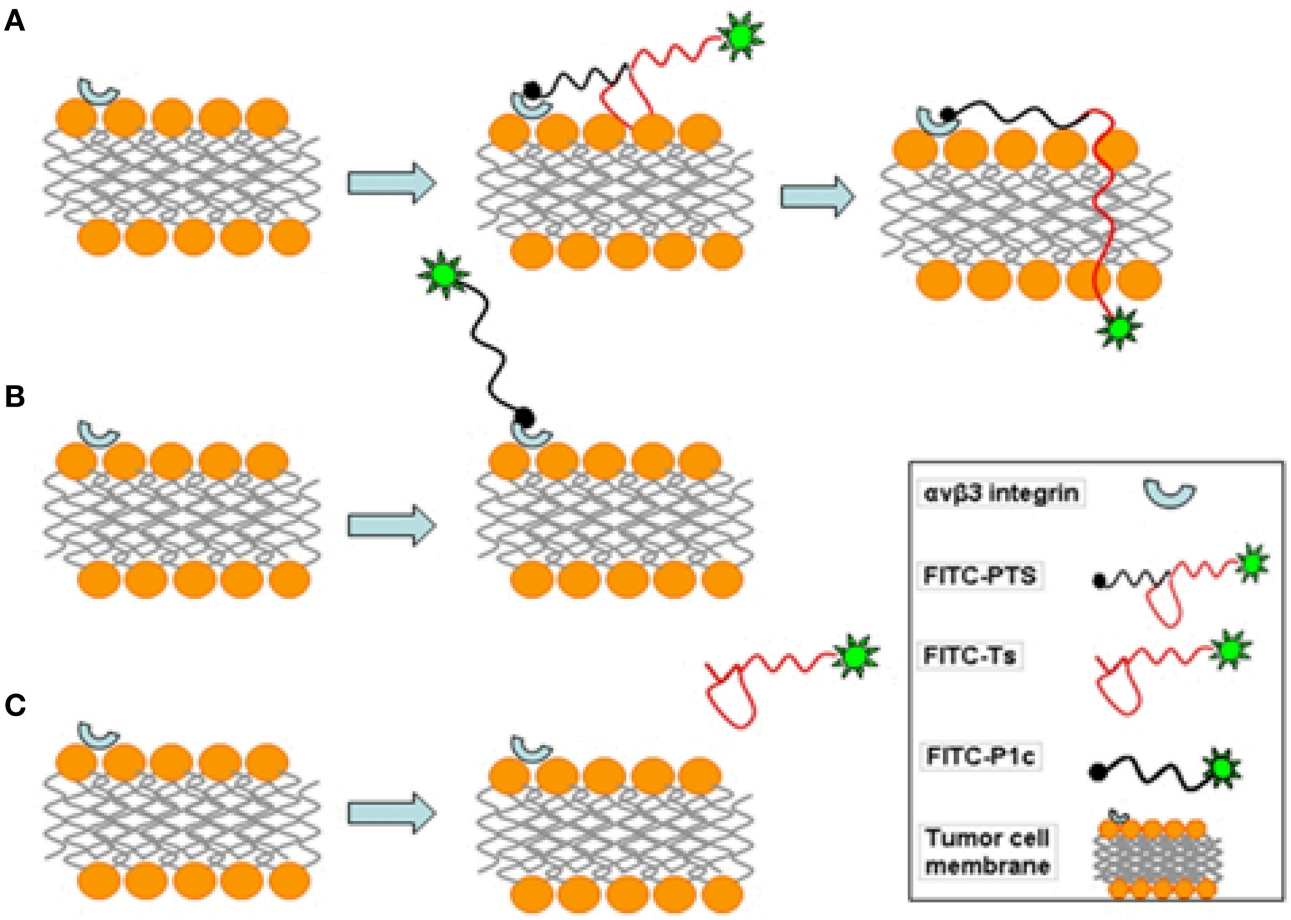
**Schematic diagram of fluorescent probes acting on MDA-MB-231 cell membrane. (A)** FITC-PTS firstly binds to integrin αvβ3 of tumor cell, and β-sheet structure tightly attaches on membrane, then FITC-labeled hydrophobic head inserts into phospholipid bilayer and rapidly penetrates into the cell. **(B)** FITC-P1c combines with integrin αvβ3 on tumor cell. **(C)** FITC-Ts has no effect on the cell membrane.

The flow cytometry and confocal microscopy results revealed that FITC-labeled PTS or P1c could sufficiently target the MDA-MB-231 cancer cells. The inhibitory effect of P1c peptide or anti-αvβ3 monoclonal antibody indicated that the targeting of FITC-PTS and FITC-P1c toward cancer cells was triggered by an initial interaction between P1c with αvβ3 located on the cell surface. HEK293 or MCF-7 cells (Figures S3, S4) lacking αvβ3 were much less attractive for P1c or PTS peptide. FITC-PTS could penetrate the cell membrane, thereby suggesting that Ts caused its hybrid capability of membrane penetration. The FITC-Ts group and the control group did not show any differences. This outcome indicated the poor membrane binding capability of Ts for MDA-MB-231 cancer cells. Overall, the results matched the speculation of the present study. The transmembrane delivery of PTS involved three steps. (1) The initial targeting of PTS onto the MDA-MB-231 cell membrane was achieved by the P1c peptide, which could be specially bound to αvβ3 that was highly expressed on the cell membrane of MDA-MB-231. (2) After the initial binding, the Ts peptide was brought to the surface of the lipid bilayer and then intercalated into the membrane after adopting a special β-sheet structure. (3) The membrane then became leaky and allowed the entrance of signal probes (Figure 8).

After being concentrated onto the surface of the cytoplasma membrane, PTS was rapidly translocated inside the cytoplasma within 30 min. This effect is significantly different from the conventional receptor mediated endocytosis, which generally takes a much longer course. A considerable number of research suggests that AMPs could rapidly intercalate the lipid membrane and make it leaky within 10 min (Yeaman and Yount, 2003). Thus, the rapid translocation across the cell membrane implies that PTS employed a mechanism similar to that of TS to penetrate the cell membrane. Such mechanism is one of the main advantages of this design.

The *in vivo* effect of PTS was further evaluated by applying it for *in vivo* imaging. NIRF imaging, as a complement to nuclear imaging methods, is a powerful tool for introvital imaging in preclinical models (Yi et al., 2014). It provides both anatomical and functional/molecular information by using exogenous fluorescent probes (Yi et al., 2014). Cy5.5 is broadly used in NIRF imaging studies because of its different characteristics, including its relative bulkiness, small Stokes shift, low signal loss, and strong anti-jamming capability (Licha et al., 2000; Hsu et al., 2006). The PTS peptide was conjugated with Cy5.5 and applied for real-time NIRF imaging.

The *in vivo* NIRF imaging studies showed that the MDA-MB-231 tumors were clearly visible with high contrast to the contralateral background at all measured time points (1–24 h) after the Cy5.5-PTS injection. Moreover, the tumor-to-normal tissue contrast ratios reached their maximum at 8 h post-injection. The probe exhibited fast tumor targeting (as early as 1 h post-injection) and markedly slower washout in tumors than in normal tissues. These results supported the preferential homing of Cy5.5-PTS to tumors and/or tumor neovasculature and its rapid accumulation in tumor tissue. Consistent with the imaging findings *in vivo* and *ex vivo*, the immunohistological examination revealed the positive expression of integrin αvβ3 in the tumor cells. In addition, the accumulation of the Cy5.5-PTS probe was positively correlated with the expression and distribution density of αvβ3 in the tumor site, Cy5.5-P1c resulted in considerably smaller contrast ratios compared with Cy5.5-PTS. This difference was consistent with the results from the confocal microscopy, which indicated that P1c could only localize the probes at the cell membrane and that Ts would help PTS penetrate into the cytoplasm and accumulate in the tumor tissue in significantly high concentrations. The immunohistological staining had been repeated for three times but not in consistent with the FACS results. The average ratio of the cells expressing αvβ3 was between 20 and 30%. It might be possible that the cells altered their expression profile after implanted into the mice. This required further exploration.

Some cyanine dyes, such as Cy5.5, have been reported to be capable of tumor accumulation without conjugation with a specific targeting molecule (Licha et al., 2000). The NIRF probes in this study might have degraded because of the dye molecules that non-specifically accumulated in the tumor. This condition may partially explain the slight tumor imaging found in the Cy5.5-Ts group. The Cy5.5-Ts and -P1c groups also served as controls, and the merit of using PTS is undeniable when comparing its effect with that of Cy5.5-Ts or -P1c. In addition, we observed a relatively higher accumulation of PTS-Cy5.5 in the liver than in other organs, which suggested hepatic elimination as the major mechanism of PTS degradation.

This study has certain limitations. First, the subcellular location of the FITC-labeled probes was investigated for 30 min only; hence, a longer investigation is needed. Second, the *in vivo* experimental data were obtained from a small number of animals (three mice for each group). Moreover, the follow-up observation was only limited to 24 h; hence, the long-term effect of the probe is still unknown. Third, the exact mechanism for PTS penetrating cells needs further studies.

PTS might be useful for drug delivery, but this design of PTS-dye is more suitable for imaging. How to combine the drug with PTS is much more complicated. Only drugs with functional groups could be linked to the peptide through chemical method. If we use a design like PTS-liposome, the transmembrane mechanism should be much different from PTS alone. So we used cy5.5 and FITC which could easily link with PTS for imaging application.

In conclusion, we designed a hybrid peptide (PTS) comprising P1c and Ts. P1c led the binding of PTS to the MDA-MB-231 cancer cell membrane, and Ts facilitated the rapid translocation of PTS across the cell membrane. PTS exhibited good specificity in the MDA-MB-231 tumor imaging via NIRF and showed great potential as a carrier in special tumor imaging.

## Author contributions

XY prepared the manuscript and did most of the *in vitro* and *in vivo* experiments and prepared the results; QQ prepared the fluorescence probe and did the circular dichroism spectroscopy assay. XF and XX helped with the result discussion and revised the manuscript; GW and NL were the group leaders offering supervision and financial support.

### Conflict of interest statement

The authors declare that the research was conducted in the absence of any commercial or financial relationships that could be construed as a potential conflict of interest.

## Abbreviations

NIRF: near-infrared fluorescence
CTGF: connective tissue growth factor
CPPs: cell penetrating peptides
AMPs: antimicrobial peptides
CLSM: confocal laser scanning microscopy
CD: Circular dichroism.
