# Supplementary material for: A Hybrid Peptide PTS that Facilitates Transmembrane Delivery and Its Application for the Rapid In vivo Imaging via Near-Infrared Fluorescence Imaging
Source: Front Pharmacol. 2016 Mar 8;7:51. doi: 10.3389/fphar.2016.00051 (PMC4782124; doi:10.3389/fphar.2016.00051)
Supplement: Supplementary file 1 [file DataSheet3.DOCX]

**
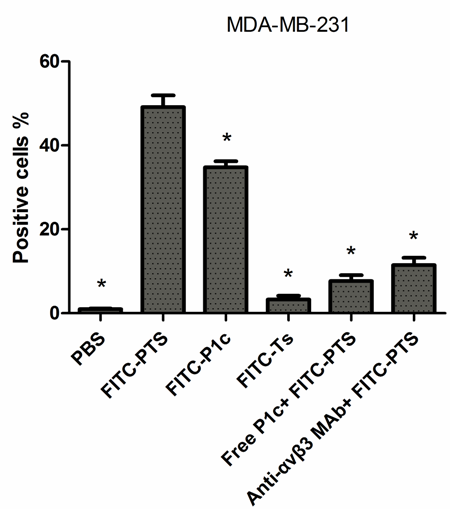
**

**Figure S1.** The positive rate of the MDA-MB-231 cells after receiving PBS or fluorescent probes by flow cytometry. *P<0.05 compared to FITC-PTS group.





**Figure S2.** The positive rate of the MDA-MB-231 cells and HEK231 cells after receiving FITC-PTS, -P1c and –Ts by flow cytometry. *P<0.05

**
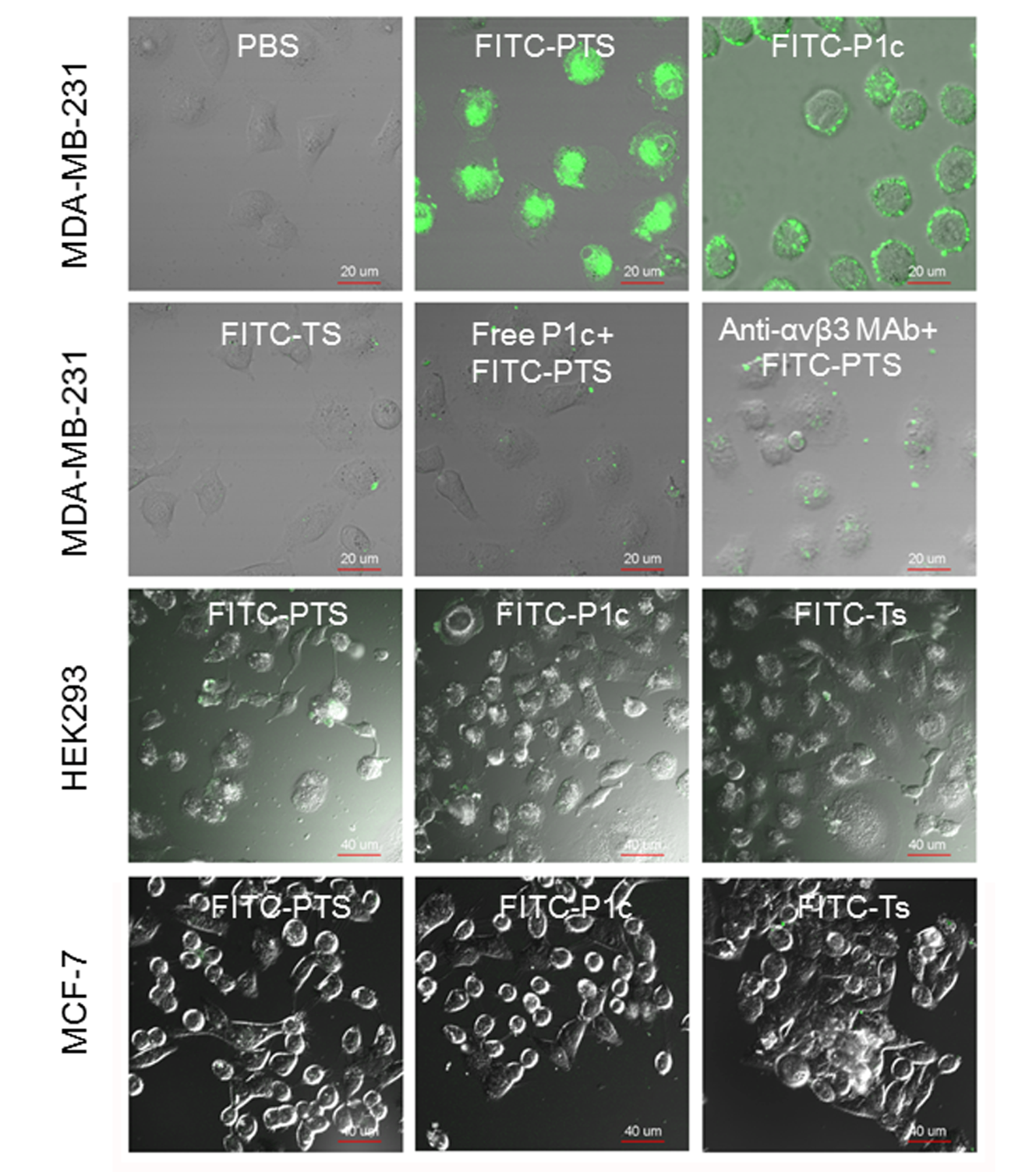
**

**Figure S3.** Cellular localization of fluorescent probes. The merged view of laser confocal florescence microscopy of MDA-MB-231, HEK293 or MCF-7 cells treated for 30 minutes with FITC-PTS, FITC-P1c , FITC-Ts , free P1c peptide followed by FITC-PTS, and anti-αvβ3 monoclonal antibody followed by FITC-PTS , separately.

**
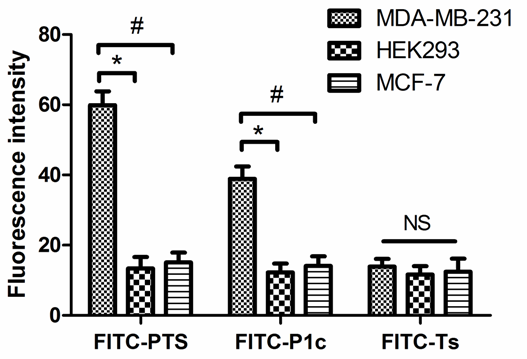
**

**Figure S4.** The fluorescent intensity of the MDA-MB-231, HEK231 and MCF-7 cells after receiving FITC-PTS, -P1c and –Ts. *,#P<0.05 compared to each other.
